# Release of *Angiostrongylus cantonensis* larvae from live intermediate hosts under stress

**DOI:** 10.1007/s00436-024-08232-y

**Published:** 2024-05-17

**Authors:** Anna Šipková, Lucia Anettová, Elena Izquierdo-Rodriguez, Vivienne Velič, David Modrý

**Affiliations:** 1 Department of Botany and Zoology, Faculty of Science, Masaryk University, Brno, Czech Republic; 2 Instituto Universitario de Enfermedades Tropicales y Salud Pública de Canarias, Universidad de La Laguna, La Laguna, Canary Islands Spain; 3 Department of Obstetrics and Gynecology, Pediatrics, Preventive Medicine and Public Health, Toxicology, Legal and Forensic Medicine and Parasitology, Universidad de La Laguna, La Laguna, Canary Islands Spain; 4 University of Veterinary Sciences Brno, Palackého tř. 1946/1, 612 42 Brno, Czech Republic; 5 Institute of Parasitology, Biology Center of Czech Academy of Sciences, České Budějovice, Czech Republic; 6 Department of Veterinary Sciences, Faculty of Agrobiology, Food and Natural Resources/CINeZ, Czech University of Life Sciences Prague, Prague, Czech Republic

**Keywords:** *Angiostrongylus cantonensis*, Stress stimuli, Larvae release, Gastropods

## Abstract

The metastrongyloid nematode *Angiostrongylus cantonensis* causes eosinophilic meningitis in a variety of homeothermic hosts including humans. Third-stage infectious larvae develop in gastropods as intermediate hosts. Humans are usually infected by intentional or incidental ingestion of an infected mollusk or paratenic host (poikilothermic vertebrates and invertebrates). The infection may also hypothetically occur through ingestion of food or water contaminated by third-stage larvae spontaneously released from gastropods. Larvae are thought to be released in greater numbers from the intermediate host exposed to stress. This study aimed to compare larval release from stressed with unstressed gastropods. Experimentally infected *Limax maximus* and *Lissachatina fulica* were exposed to a stress stimulus (shaking on an orbital shaker). The mucus was collected before and after the stress and examined microscopically and by qPCR for the presence of *A. cantonensis* larvae and their DNA. In the case of *L. maximus*, no larvae were detected microscopically in the mucus, but qPCR analysis confirmed the presence of *A. cantonensis* DNA in all experimental replicates, without clear differences between stressed and non-stressed individuals. In contrast, individual larvae of *A. cantonensis* were found in mucus from *Li. fulica* after stress exposure, which also reflects an increased number of DNA-positive mucus samples after stress. Stress stimuli of intensity similar to the transport or handling of mollusks can stimulate the release of larvae from highly infected intermediate hosts. However, these larvae are released in small numbers. The exact number of larvae required to trigger neuroangiostrongyliasis is unknown. Therefore, caution is essential when interacting with potential intermediate hosts in regions where *A. cantonensis* is endemic.

## Introduction

*Angiostrongylus cantonensis* is an invasive parasitic metastrongyloid nematode (Chen 1935; Cowie 2013a). Its heteroxenous life cycle involves rodents, especially *Rattus* spp., as definitive hosts. First-stage larvae (L1) are released in feces and ingested by gastropods (intermediate hosts), where they develop into third-stage larvae (L3) infectious for the definitive host (Cowie 2013b). Third-stage larvae can further survive in paratenic hosts, such as frogs or lizards among various other taxa (Anettová et al. 2022; Ash 1968; Rosen et al. 1962; Turck et al. 2022). This complex cycle can lead to accidental infections of avian or mammalian hosts by various infection pathways. In these aberrant hosts, however, the parasite causes severe neurological disease, known as neuroangiostrongyliasis or ocular infections (Diao et al. 2011; Duffy et al. 2004; Lunn et al. 2012; Monks et al. 2005; Wang et al. 2008; Wright et al. 1991).

In humans, a typical way to acquire infection is by ingestion of raw or undercooked intermediate hosts (gastropods) or paratenic hosts (reptiles, amphibians) that contain L3 (Anettová et al. 2022; Federspiel et al. 2020; Lai et al. 2007; Pai et al. 2013; Turck et al. 2022); additionally, larvae are able to survive for a limited period of time in other invertebrate hosts (crustaceans, centipedes). Another yet hypothetical pathway of infection is the ingestion of vegetables or water contaminated with L3 released from intermediate hosts (Chen and Alicata 1964; Cowie 2013b). While the liberation of L3 from dead (e.g., drowned) gastropods is well documented (Howe et al. 2019; Modrý et al. 2021), the spontaneous emergence of infective larvae from live hosts and factors impacting this phenomenon remains the most neglected part of *A. cantonensis* transmission cycles (Heyneman and Lim 1967; Kramer et al. 2018). Recently, Rollins et al. (2023) demonstrated an increased number of L3 emerging from the terrestrial gastropod *Parmarion martensi* in response to elevated temperature, molluscicide, and mechanical disturbance.

Some human cases of neuroangiostrongyliasis have been causally connected to ingestion of raw intermediate or paratenic hosts; however, many cases remain without a confirmed infection source (Federspiel et al. 2020; Pandian et al. 2023; Wang et al. 2008). The clinical cases in children in an endemic area on the Indian Ocean island of Mayotte were tentatively associated with the handling of presumably infected giant African snails, *Lissachatina fulica* (Epelboin et al. 2016).

*Lissachatina fulica* is an invasive snail species that has successfully colonized tropical regions around the world (Ayyagari and Sreerama 2017; Silva et al. 2022). Because of its size, attractive appearance, and abundance in peridomestic spaces, this species is commonly handled by humans. We hypothesize that manipulation or unwanted transporting of mollusks represents a stress stimulus that can increase the release of L3 and increase the chance of accidental infection in humans. This study on artificially infected stressed giant African snails and European slugs further corroborates the active emergence of *A. cantonensis* infective larvae from their molluscan hosts.

## Materials and methods

### Animals

**Fig. 1:**
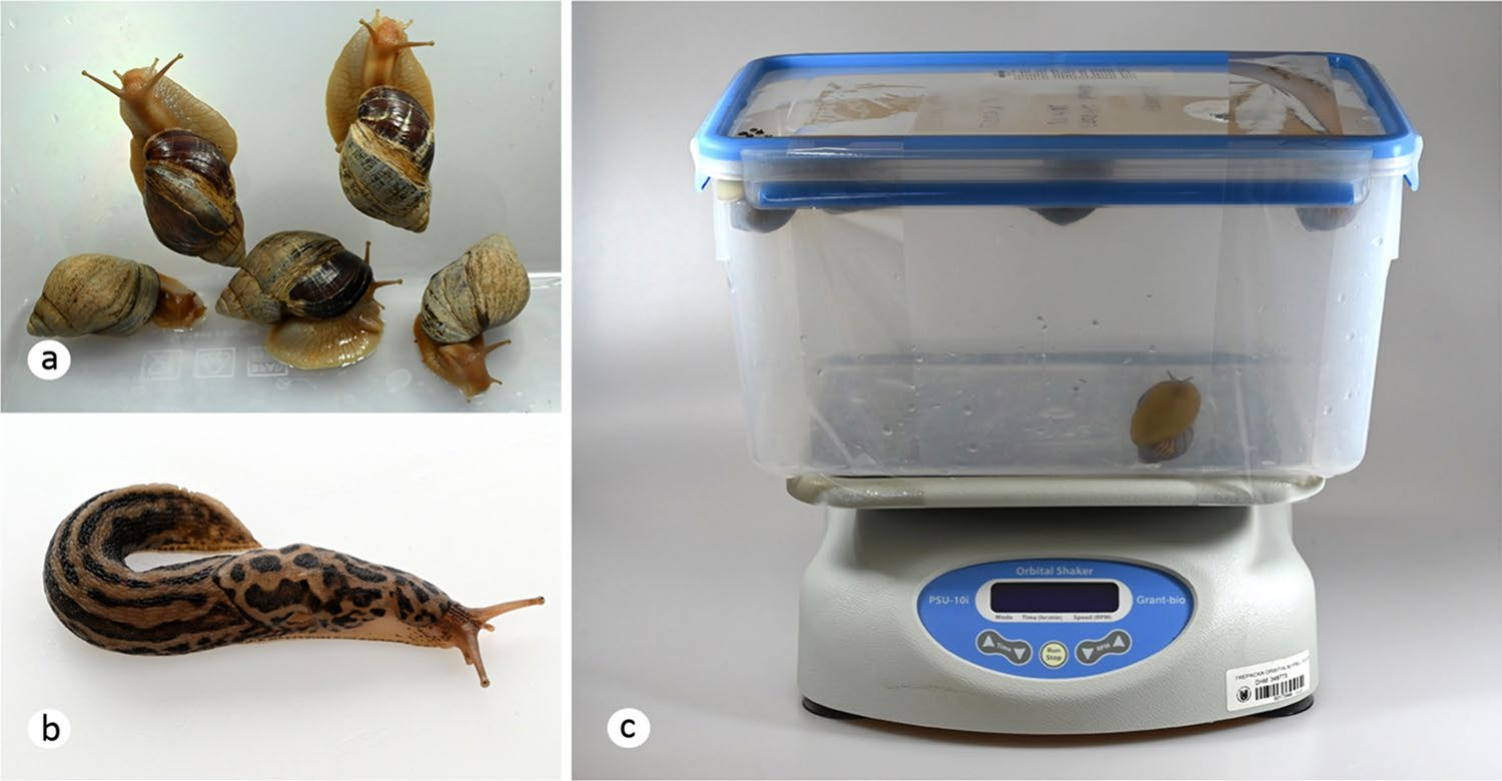
**a** *Lissachatina fulica* in an experimental box during the non-stress phase of the experiment (in the stress phase, snails were partly retracted into the shell); **b** *Limax maximus* during the non-stress phase of the experiment; **c** experimental design of the stress stimulation: box with experimental *L. fulica* attached to the orbital shaker

The experimental field isolate of *A. cantonensis* was brought from Fatu Hiva, French Polynesia in 2017. The life cycle was maintained in laboratory conditions (Modrý et al. 2021) using laboratory rats (*Rattus norvegicus*, Wistar strain) and experimental laboratory-reared gastropods (*Lissachatina fulica*); worms were identified as *A. cantonensis* clade 2 based on cox1 sequences (Červená et al. 2019). Two species of experimental gastropods were selected. *Lissachatina fulica* is an invasive species commonly associated with human infections by *A. cantonensis*; the great grey slug, *Limax maximus*, is a broadly distributed European species proven as a suitable intermediate host of *A. cantonensis* in temperate Europe (Anettová et al. 2022; Lima et al. 2020). Captive subadult *L. fulica* (Fig. 1a) were purchased from a private breeder, and *L. maximus* (Fig. 1b) were collected from a garden near Brno in the autumn of 2021. The individuals of each species were divided into plastic boxes each containing 5–10 individuals (85 *L. maximus* and 31 *L. fulica*) and provided with coconut soil, shelters, and moss. Approximately 1 g of rat feces was added to each box each day for 2 weeks. Baerman’s method was used to determine the number of L1 per gram of rat feces. As a result, mollusks of each box received approximately 40,000 first-stage larvae within 2 weeks. *Lissachatina fulica* typically consumed the entirety of the feces provided, whereas *L. maximus* was less inclined to consume the feces*.* After the infection period, mollusks were divided into 7 experimental groups and left for 4 weeks to allow the L1 to develop into L3.

In total, 85 adult *L. maximus* slugs of an average weight of 2.2 g (± 0.6 g) were divided into 3 experimental groups (M1—22 individuals, M2—27 individuals, M3—30 individuals) and a positive control group of 6 individuals. During the development period, some individuals in the experimental groups of *L. maximus* died, and therefore, only 22 (group M1) and 27 (group M2) of the original 30 individuals in each group remained. *Lissachatina fulica* snails (31 in total) with an average weight of 37.3 g (± 1.8 g) were divided into 2 experimental groups (G1—12 individuals; G2—12 individuals) and a positive control group of 7 individuals. Individual experimental groups consisting of *L. maximus* (M1, M2, M3) and *L. fulica* (G1, G2) were subjected to two experimental phases: a non-stress phase (without shaking) and a stress phase (with shaking). Positive controls, which were infected and unshaken served as confirmation of the presence of L3 at the end of the experiment.

### The emergence of A. cantonensis third-stage larvae

All gastropods were first rinsed with tap water to remove the residual substrate, and all individuals of the given group were placed in a separate plastic box with a perforated lid. In the first phase, the mollusks were left in boxes without any stress stimuli for 4 h at 22 °C and exposed to daylight. After this period, individuals of *L. maximus* were placed in 50-ml Falcon tubes with 15 ml of tap water and gently washed for 30 s to collect the mucus produced. Considering their larger size, *L. fulica* individuals were placed in a funnel and washed with 15 ml of water, which was collected directly into 50-ml Falcon tubes. The mucus from the boxes in which the experimental groups were left was washed with 50 ml of water and collected. The samples were centrifuged (1 min, 1500 g) and stored for subsequent DNA extraction and qPCR analysis. In the second phase, each box with experimental mollusks was fixed on an orbital shaker with adhesive tape and shaken for 4 h at 130 rpm (Fig. 1c). The mucus produced from individuals and from boxes was washed out in a similar way as described above.

### Isolation of the DNA from mucus and qPCR analysis

The centrifuged sediment from the mucus samples was used for the DNA extraction with a DNEasy Blood and Tissue (Qiagen, Germany) extraction kit. The procedure was optimized for L3 of *A. cantonensis* when the pre-lyse phase was extended overnight. A species-specific qPCR assay on LightCycler 480 was used to detect the presence of *A. cantonensis* DNA in the mucus (Sears et al. 2021); the reagents and volumes used are described in Table 1. The cycling conditions of the total number of 40 cycles were set as follows: 95 °C for 20 s, 40 °C for 1 s, and 60 °C for 20 s. DNA from a single L3 isolated by the same process as the mucus samples, which was 100 × diluted, was used as a positive control for qPCR analysis. PCR water was used as a negative control. The samples were analyzed in duplicates (Ct values not exceeding 35 were determined as positive).

**Table 1:** Reagents and volumes used for qPCR diagnostics

Reagents	Volumes per well (µl)
DNA, DNase, and RNase-free water	6.2
2 × MasterMix (IDT Prime time gene expression master)	10
10 µM probe (PrimeTime Eco Probe 5′ 6-FAM/ZEN/3′ IBFQ, /56-FAM/ACA TGA AAC/ZEN/ACC TCA AAT GTG CTTCGA/31ABkFQ/)	00.2
10 µM forward primer (AAA CTG TTG CTT TCG AAG CTA TG)	0.8
10 µM reverse primer (GCG CAA ATC TGA CGT TCT TG)	0.8
DNA template	2
Total volume	20

### Artificial digestion

After the stress phase of the experiment was complete, all mollusks (except group M3) were sacrificed by decapitation and artificially digested in order to facilitate determining the total number of L3 in individual slugs and snails. Group M3 was not sacrificed and was utilized for additional purposes beyond the scope of the experiment. The whole bodies (without shells in the case of *L. fulica*) were cut and fully digested in a digestion fluid (3 g pepsin, 28 ml 25% HCl in a total volume of 1 l of distilled water) on a magnetic stirrer set to 600 rpm at 37 °C for 2 h. The digested tissue was transferred through a sieve into 50-ml Falcon tubes and centrifuged (1 min, 1500 g). The sediment was gradually transferred to Petri dishes, and the L3 were counted under a light microscope (magnification 40 ×). Positive controls of *L. maximus* and *L. fulica* were processed by the same method and the presence of L3 in the tissues of infected mollusks was microscopically confirmed.

### Statistics

GraphPad Prism 9.5.1. was used to perform statistical analysis using Fisher’s exact test. Two groups were tested: *L. maximus* (79 individuals) and *L. fulica* (24 individuals). Means, standard deviations, and percentages of positive samples were calculated using Microsoft Excel to further characterize the data.

## Results

L1 developed into L3 in the tissues of all infected individuals of *L. fulica* and *L. maximus*. The average number of L3 per slug in group M1 was 18 (± 15) and in group M2 was 18 (± 16), whereas, in *L. fulica*, the average number in group G1 was 7354 (± 5428) and in group G2 was 14620 (± 8547). The average number of larvae in the positive control groups was 17 (± 15) for *L. maximus* and 11,145 (± 7981) for *L. fulica*.

**Fig. 2:**
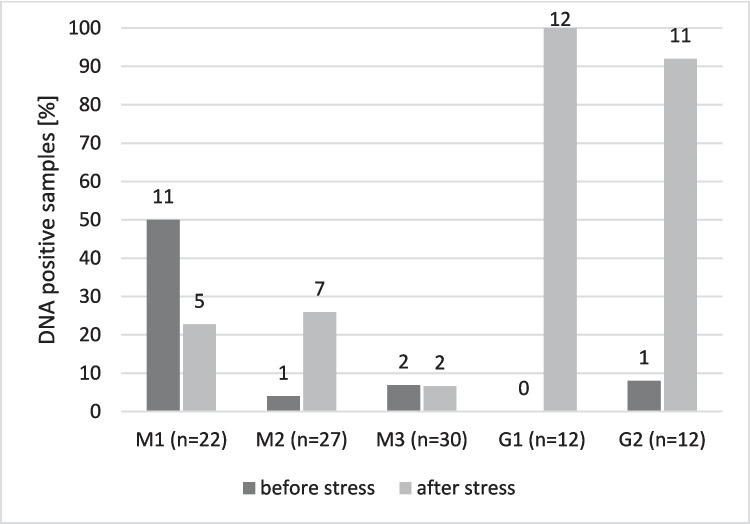
Number and percentage of samples positive for DNA of *A. cantonensis* by qPCR in the experiment with *Limax maximus* and *Lissachatina fulica* before and after stress stimulus (DNA extracted from mucus collected from each individual); M1, M2, M3 = experimental groups of *L. maximus*; G1, G2 = experimental groups of *L. fulica.* The numbers above the bars correspond to the number of positive DNA samples in experimental groups

L3 of *A. cantonensis* were not detected in the mucus from *L. maximus* or from the boxes contaminated with mucus. However, the presence of *A. cantonensis* DNA was confirmed by qPCR analysis. An increase in the number of positive samples after shaking was observed only in group M2; no change was observed in group M3, while the number of positive samples after stress decreased in group M1 (Fig. 2). The difference between the number of DNA-positive samples before and after shaking was not statistically significant (*p* = 1.0000). The DNA positivity was confirmed in the mucus collected from the boxes before and after the stress stimulus in groups M2 and M3. In group M1, the presence of DNA in the mucus from the box was not confirmed.

Out of 24 *L. fulica* examined individually, only a single live *A. cantonensis* larva was detected (individual from G1, after stress); 4 additional live larvae were captured in the mucus collected from the G2 box after stress. The difference in the number of DNA-positive samples from individuals before and after stress stimulus was statistically significant (*p* = 0.0001). The number and percentage of positive individual samples before and after the stress stimulus are shown in Fig. 2. The presence of DNA in the mucus was confirmed before and after the stress stimulus in the G1 box but only after the stress stimulus in the G2 box.

## Discussion

Neuroangiostrongyliasis in mammals, including humans, is primarily associated with the ingestion of raw intermediate hosts (Cowie 2013b). Ingestion can be intentional as is usual in countries where raw gastropods are consumed (Eamsobhana 2014). Several cases of infection have resulted from the intentional eating of raw snails or slugs as a bet or dare (Cowie et al. 2023; Kwon et al. 2013). Transmission can also occur through the ingestion of freshwater shrimp, amphibians, or reptiles (Anettová et al. 2022; Turck et al. 2022). However, inadvertent ingestion of L3 may also occur with raw vegetable products, on which small snail or slug individuals might be overlooked (Ash 1976; Tsai et al. 2004; Waugh et al. 2005). Finally, it is possible that humans may get infected by ingestion of L3 liberated from an intermediate host into drinking water or by mucus that can remain on food or hands (Ash 1976; Heyneman and Lim 1967; Modrý et al. 2021; Qvarnstrom et al. 2007).

The ability of infective larvae of *A. cantonensis* to escape from dead intermediate hosts is notoriously known, and such larvae can survive in aquatic environments for almost a month (Crook et al. 1971; Modrý et al. 2021). Survival of L3 in drinking water reservoirs has been theorized as a cause of cases of eosinophilic meningitis in humans in Hawaii (Howe et al. 2019). A similar release of L3 from dead experimentally infected *Cornu aspersa* has been described in metastrongyloid nematodes of carnivores, such as *Aelurostrongylus abstrusus* and *Troglostrongylus brevior* (Giannelli et al. 2015). Released L3 can be further transferred horizontally between individual mollusks in a process referred to as intermediesis (Colella et al. 2015; Modrý et al. 2021).

However, L3 can also spontaneously escape from live intermediate hosts, as demonstrated in *Angiostrongylus vasorum*, *Aelurostrongylus abstrusus*, *Crenosoma vulpis*, *Oslerus rostratus*, and *Troglostrongylus wilsoni* from experimentally infected gastropods, *Biomphalaria glabrata* and *Limax maximus* (Barçante et al. 2003; Conboy et al. 2017; Robbins et al. 2021). Reports confirming the spontaneous liberation of *A. cantonensis* L3 from live intermediate hosts are sporadic. In some cases, the presence of a low number of L3 in the mucus was confirmed (Ash 1976; Heyneman and Lim 1967; Kramer et al. 2018; Qvarnstrom et al. 2007; Waugh et al. 2016), while Campbell and Little (1988) did not confirm the release of L3 from *Limacus flavus*.

Only two studies have dealt with stressors impacting larval release. In the case of the related species *A. vasorum*, Barçante et al. (2003) confirmed that L3 were shed from *B. glabrata* after exposure to elevated temperature or light. Recently, Rollins et al. (2023) demonstrated that *A. cantonensis* larvae were released from stressed semi-slugs (*P. martensi*); however, spontaneous liberation of larvae without stress stimuli was not observed. The design of our research further extends the latter study. Besides the detection of parasite DNA by qPCR, we also investigated the presence of live larvae, using *L. fulica* as a previously hypothesized source of human infections, complemented with experimental *L. maximus*, a European slug species proven as an intermediate host. Mechanical shaking, similar to that performed by Rollins et al. (2023), was used to mimic mollusk handling or transport, as infection by larvae liberated this way from infected intermediate hosts was previously hypothesized (Asato et al. 2004; Wan and Weng 2004).

In the case of *L. maximus*, our visual observations did not detect larvae in the mucus either before or after the stress exposure, supporting the previous negative result of Campbell and Little (1988) with closely related slugs. However, the presence of *A. cantonensis* DNA in the mucus was confirmed both before and after the stress exposure, which may indicate the release of L3 in very low numbers. This corresponds to the overall lower number of L3 found in *L. maximus* tissues, which may originate from the reduced tendency of *L. maximus* to eat rat feces. Additionally, the mucus produced was not easy to collect, as it was firmly attached to the surface of both the gastropods and the walls of the boxes, so some material may have been lost during collection.

In contrast, we confirmed the presence of live L3 of *A. cantonensis* in the mucus of *L. fulica*, but only after the stress exposure. Also, the presence of *A. cantonensis* DNA in mucus samples showed a significant increase in the number of positive samples in stressed individuals of *L. fulica*, which is consistent with the results of Rollins et al. (2023). However, the numbers of detected larvae were very low compared to the mucus larval load extrapolated from Ct values (qPCR results) by Rollins et al. (2023). Experiments focused on the release of *A. vasorum* L3 from aquatic snails also demonstrated higher numbers (Barçante et al. 2003). In the case of experimental *L. fulica*, the absolute numbers of L3 detected in tissue were much higher than in *L. maximus*, ranging around 10^4^, similar to the highest infection intensities observed in naturally infected *L. fulica* (Tesana et al. 2009). The dependence of higher larval excretion on infection intensity in intermediate hosts was confirmed by Rollins et al. (2023), and this may have been a key factor for the release of the L3 in our experimental *L. fulica*.

Stress stimuli of intensity comparable to mollusk transportation or handling could elicit a larval release from heavily infected individuals, though in small numbers. The invasive giant African snail, *L. fulica*, is common in peridomestic environments both in rural and urban areas in many tropical and subtropical regions of the world, and frequent contact with these snails in areas endemic to *A. cantonensis* has been mentioned as one of the possible risk factors (Epelboin et al. 2016).

Results of our study indicate that mild stress stimuli do not significantly enhance the L3 release, thus not heightening the risk of human infection. However, our research was confined only to two species of intermediate hosts and to a single type of stress stimulus. The outcomes may differ in other intermediate host species and may be influenced by a type of stressor or by its delayed effect. Although simple handling of mollusks is unlikely to cause significant larval release, caution should be exercised when interacting with intermediate hosts in areas endemic to *A. cantonensis*, considering also the fact that a precise number of larvae needed to cause disease manifestations remains unknown.

## Data Availability

All data obtained in this study are included in the article.
